# Effects of Exposure to Intermittent versus Continuous Red Light on Human Circadian Rhythms, Melatonin Suppression, and Pupillary Constriction

**DOI:** 10.1371/journal.pone.0096532

**Published:** 2014-05-05

**Authors:** Ivan Ho Mien, Eric Chern-Pin Chua, Pauline Lau, Luuan-Chin Tan, Ivan Tian-Guang Lee, Sing-Chen Yeo, Sara Shuhui Tan, Joshua J. Gooley

**Affiliations:** 1 Graduate School for Integrative Sciences and Engineering, National University of Singapore, Singapore, Singapore; 2 Program in Neuroscience and Behavioral Disorders, Duke-NUS Graduate Medical School, Singapore, Singapore; 3 Division of Sleep and Circadian Disorders, Departments of Medicine and Neurology, Brigham and Women's Hospital, Boston, Massachusetts, United States of America; 4 Division of Sleep Medicine, Harvard Medical School, Boston, Massachusetts, United States of America; University of Lübeck, Germany

## Abstract

Exposure to light is a major determinant of sleep timing and hormonal rhythms. The role of retinal cones in regulating circadian physiology remains unclear, however, as most studies have used light exposures that also activate the photopigment melanopsin. Here, we tested the hypothesis that exposure to alternating red light and darkness can enhance circadian resetting responses in humans by repeatedly activating cone photoreceptors. In a between-subjects study, healthy volunteers (n = 24, 21–28 yr) lived individually in a laboratory for 6 consecutive days. Circadian rhythms of melatonin, cortisol, body temperature, and heart rate were assessed before and after exposure to 6 h of continuous red light (631 nm, 13 log photons cm^−2^ s^−1^), intermittent red light (1 min on/off), or bright white light (2,500 lux) near the onset of nocturnal melatonin secretion (n = 8 in each group). Melatonin suppression and pupillary constriction were also assessed during light exposure. We found that circadian resetting responses were similar for exposure to continuous versus intermittent red light (*P* = 0.69), with an average phase delay shift of almost an hour. Surprisingly, 2 subjects who were exposed to red light exhibited circadian responses similar in magnitude to those who were exposed to bright white light. Red light also elicited prolonged pupillary constriction, but did not suppress melatonin levels. These findings suggest that, for red light stimuli outside the range of sensitivity for melanopsin, cone photoreceptors can mediate circadian phase resetting of physiologic rhythms in some individuals. Our results also show that sensitivity thresholds differ across non-visual light responses, suggesting that cones may contribute differentially to circadian resetting, melatonin suppression, and the pupillary light reflex during exposure to continuous light.

## Introduction

Classical visual photoreceptors (i.e., rods and cones) are required for sight, but not for non-visual light responses including entrainment of circadian rhythms, inhibition of pineal gland synthesis of the hormone melatonin, and the pupillary light reflex [1]–[3]. In the absence of rod and cone function, these light responses are mediated by intrinsically photosensitive retinal ganglion cells (ipRGCs) that contain the photopigment melanopsin [4], [5]. Melanopsin-containing ipRGCs project directly to the suprachiasmatic nucleus (SCN) in the hypothalamus to regulate light resetting of circadian rhythms and melatonin suppression, and to the pretectal area to mediate light-induced pupillary constriction [6], [7]. The ipRGCs respond preferentially to short-wavelength light in the blue portion of the visual spectrum (λ_max_ = ∼480 nm) [8], [9], hence non-visual light responses are most sensitive to blue light in blind humans or sightless mice with intact function of the inner retina [3], [4], [10], [11].

Melanopsin cells respond directly to light but can also be activated extrinsically by light stimulation of rod and cone photoreceptors in the outer retina [8], [9], [12]. Consequently, non-visual light responses remain intact even when melanopsin is rendered dysfunctional [13], [14]. In melanopsin null mice, exposure to light during the subjective night induces circadian phase resetting and inhibits mRNA levels for arylalkylamine *N*-acetyltransferase [5], the rate-limiting enzyme of the melatonin biosynthetic pathway. Circadian phase resetting, melatonin suppression, and pupillary light responses are only eliminated when rod-cone and melanopsin signaling pathways are disrupted simultaneously [4], [5]. These studies demonstrate that classical visual photoreceptors can drive non-visual light responses, but the role of cone photoreceptors remains controversial. Mice that lack rod and melanopsin function, but have intact cones, do not show reliable circadian photic entrainment [15], [16]; however, phase shift responses to short-duration light stimuli (1 or 5 min) are attenuated in mice that lack functional mid-wavelength-sensitive cones (M-cones) [17]. In humans, the sensitivity of melatonin suppression to 555-nm green light, which stimulates the three-cone photopic system maximally, decreases gradually over time during exposure to continuous light [18]. Similarly, the magnitude of pupillary constriction decreases gradually during exposure to green light [10], and there is a short-wavelength shift in spectral responses over time [10], [19]. Together, these studies suggest that cone photoreceptors contribute to non-visual light responses at the beginning of light exposure, but their role diminishes relative to melanopsin with increasing duration of light.

In principle, the contribution of cones to non-visual light responses could be enhanced by giving intermittent dark pulses that allow cone photoreceptors the opportunity to dark adapt between light pulses. As shown for circadian phase resetting in mice [15], and the pupillary light reflex in humans [10], exposure to alternating long-wavelength light and darkness can increase some non-visual light responses by activating cone photoreceptors repeatedly. In the present study, we therefore tested the hypothesis that human volunteers exposed to intermittent red light and darkness would show larger circadian phase shifts, and stronger suppression of melatonin, relative to individuals exposed to continuous red light.

## Materials and Methods

### Ethics Statement

Study procedures were approved by the SingHealth Centralized Institutional Review Board and were in compliance with the Declaration of Helsinki. Written informed consent was obtained from all participants.

### Subjects

Ethnic-Chinese males (n = 24) aged 21–28 years were recruited from the general population. Subjects were healthy and non-smokers based on their responses on a structured health questionnaire. Chronotype was assessed using the Horne-Östberg Morningness-Eveningness Questionnaire (MEQ), and sleep quality in the month prior to the study was assessed using the Pittsburgh Sleep Quality Index (PSQI). Definite morning or evening types were excluded (MEQ score <31 or >69), as were individuals who reported poor sleep quality (PSQI score >5). The Ishihara Color Blindness Test was used to select for participants with normal color vision. Additional eligibility criteria included no history of shift work and no travel across time zones in the 3 weeks preceding the study. Participants were required to keep a regular sleep-wake schedule for at least 1 week before the laboratory study, with 8 h of time in bed for sleep each night. Compliance was verified by sleep diaries and wrist actigraphy (Actiwatch-L or Actiwatch 2, Philips Respironics, The Netherlands). Subjects were also instructed to abstain from caffeine, alcohol, and nicotine in the week prior to the laboratory study.

### Protocol

Participants lived individually in a research suite for 6 days at the Chronobiology and Sleep Laboratory, Duke-NUS Graduate Medical School Singapore. The research suite consisted of a time-free environment without windows. Participants arrived in the afternoon and were oriented to study procedures before going to bed at their regular pre-study bedtime. After 8 h of time in bed for sleep, participants were kept awake for 26 h using constant routine (CR) procedures as previously described [20]. At the end of the CR procedure, subjects were given an 8-h opportunity for sleep during the daytime. Subjects then awoke in the evening and underwent a 6-h light exposure procedure starting one hour before their habitual bedtime. Participants were assigned randomly to receive continuous red light, intermittent red light and darkness, or bright white light (details provided below). After 16 h of being awake, subjects were given 8 h of time in bed for sleep. Upon waking in the evening, participants underwent a second 26-h CR procedure. Subjects were then given a 12-h recovery sleep period and were discharged from the study on the following day.

### Light Exposure

The 6-h light exposure procedure comprised 6 cycles of a 50-min fixed gaze period and a 10-min free gaze period. During the fixed gaze period of each cycle, participants were seated with their head position fixed by a chinrest. Narrow-bandwidth 631-nm red light was delivered using a modified Ganzfeld dome, with a light-emitting diode (LED) as the light source (Nichia Corporation, Tokushima, Japan). During the free gaze period, subjects remained seated but removed their head from the dome and were free to look elsewhere. Since the light source remained on and the room was otherwise dark, subjects were exposed to less light during free gaze periods relative to fixed gaze periods. We did not attempt to estimate corneal light exposure during free gaze periods, however, as the head position was not fixed. Intermittent light exposure was achieved utilizing an electronic function generator (Keithley Instruments Inc., Cleveland, Ohio). Due to the oscillating frequency being limited to a minimum step of 0.001 Hz, intermittent light pulses were delivered at a frequency of 0.008 Hz (62.5 s on, 62.5 s off), approximating a 1-min on/off pattern. Light intensity was measured before the light exposure session and during each free gaze period using a portable radiometer (ILT1400 or ILT1700, International Light Technologies, Peabody, Massachusetts) with the sensor placed at the level of the subjects' eyes during light exposure. Irradiance was 13 log photons cm^−2^ s^−1^ for each of the red light exposure conditions, and in the bright white light condition (2,500 lux), light was delivered using a wall-mounted panel consisting of 11 fluorescent light tubes (Lumilux Cool White, 4000K; Osram, Munich, Germany).

### Pupillometry

Subjects wore a pupillometric ocular tracking device (ISCAN Inc., Woburn, Massachusetts) during the light exposure procedure. Pupil diameter of the left eye was obtained using an infrared light emitter and camera mounted on a headband, with data collected at a rate of 120 samples per second.

### Melatonin and Cortisol

Saliva samples were collected hourly during CR procedures, and twice hourly during the light exposure session using Salivette tubes (Sarstedt AG & Co., Germany). Melatonin (MLT) and cortisol (Cort) concentrations were determined by ELISAs (IBL International GmbH, Hamburg, Germany; and NovaTec Immundiagnostica GmbH, Dietzenbach, Germany respectively), using kit instructions provided by the manufacturers. A single ELISA plate was used for each subject, and the median intra-assay coefficients of variation for melatonin and cortisol assays were 8% and 15%, respectively.

### Temperature

Core body temperature (CBT) was collected continuously using a VitalSense temperature sensor that participants ingested at bedtime prior to each CR procedure. Forehead skin temperature (FST) was collected using a sensor applied to the forehead using an adhesive patch. Data were collected every minute using a VitalSense Integrated Physiologic Monitor (Mini Mitter Inc., Bend, Oregon) placed near the participant in bed. FST data were not available for 1 participant exposed to bright white light due to data acquisition problems.

### Heart Rate

The electrocardiogram was recorded continuously with a single-channel modified V5 lead. Data were acquired using a portable Grass Comet polysomnographic system (Astromed, West Warwick, Rhode Island). Heart rate (HR) was determined from the R-R interval time series derived from automatic QRS peak detection using a Hilbert transform-based method as previously described [20].

### Data Analysis

To assess circadian phase, physiologic rhythms during each CR procedure were fitted using a harmonic regression model with correlated noise [21]. Curve fitting for hormones and CBT were performed on data collected hourly and on a per-minute basis, respectively, whereas FST data were binned at hourly intervals using the median, and HR data were binned at 10-min intervals using the median in order to smooth the time-series. Circadian phase was assessed using the fitted minima of 2-harmonic regressions for CBT, FST, and HR; the maximum of a 2-harmonic regression for Cort; and the maximum of a 3-harmonic regression for MLT. In some individuals, the time-course of MLT (n = 3), Cort (n = 1), or HR (n = 3) did not show a detectable rhythm during one or both CR procedures; in these cases, a reliable phase estimate could not be determined and data were hence excluded from group analyses.

Circadian phase shift responses were modeled as the sum of fixed and random effects using a linear mixed-effects model (IBM SPSS Statistics, IBM Corp., Armonk, New York). The model included a normally distributed random intercept to represent between-subject differences around a fixed intercept, and a normally distributed error for within-subject differences across phase shift markers. Light exposure condition (continuous red light, intermittent red light, and bright white light) and physiologic measures (MLT, Cort, CBT, FST, and HR) were modeled as fixed effects. Phase shift responses between groups and physiologic measures were compared using an *F*-test, with multiple comparisons performed using Fisher's Least Significant Difference (LSD) test. To complement these analyses, we also performed simple one-way ANOVA to compare phase resetting responses between groups for each physiologic measure. In cases where data did not pass a test for normality, we performed a Kruskal-Wallis ANOVA on ranks. ANOVA and pairwise multiple comparison procedures were performed using SigmaPlot 11.0 software (Systat Software, Inc., San Jose, CA).

Melatonin suppression was determined by comparing the area under the curve (AUC) of the melatonin profile during light exposure with the AUC during the preceding constant routine at the same relative clock times. In 2 participants, melatonin levels during the light exposure session could not be determined due to problems with saliva sampling. Percentage melatonin suppression was compared between groups using one-way ANOVA, with pairwise multiple comparisons were performed using Fisher's LSD test.

Pupillometric data during the first fixed gaze period of the light exposure (i.e., across 50 min) were processed to remove blink artifacts and binned at intervals of 15.625 s, corresponding to one-quarter of an intermittent lights-on pulse. In 2 participants, pupillary constriction could not be determined reliably due to artifacts in the recording. Pupil diameter during the light exposure was expressed as a percentage of each participant's pupil size measured in darkness.

## Results

**Figure 1:**
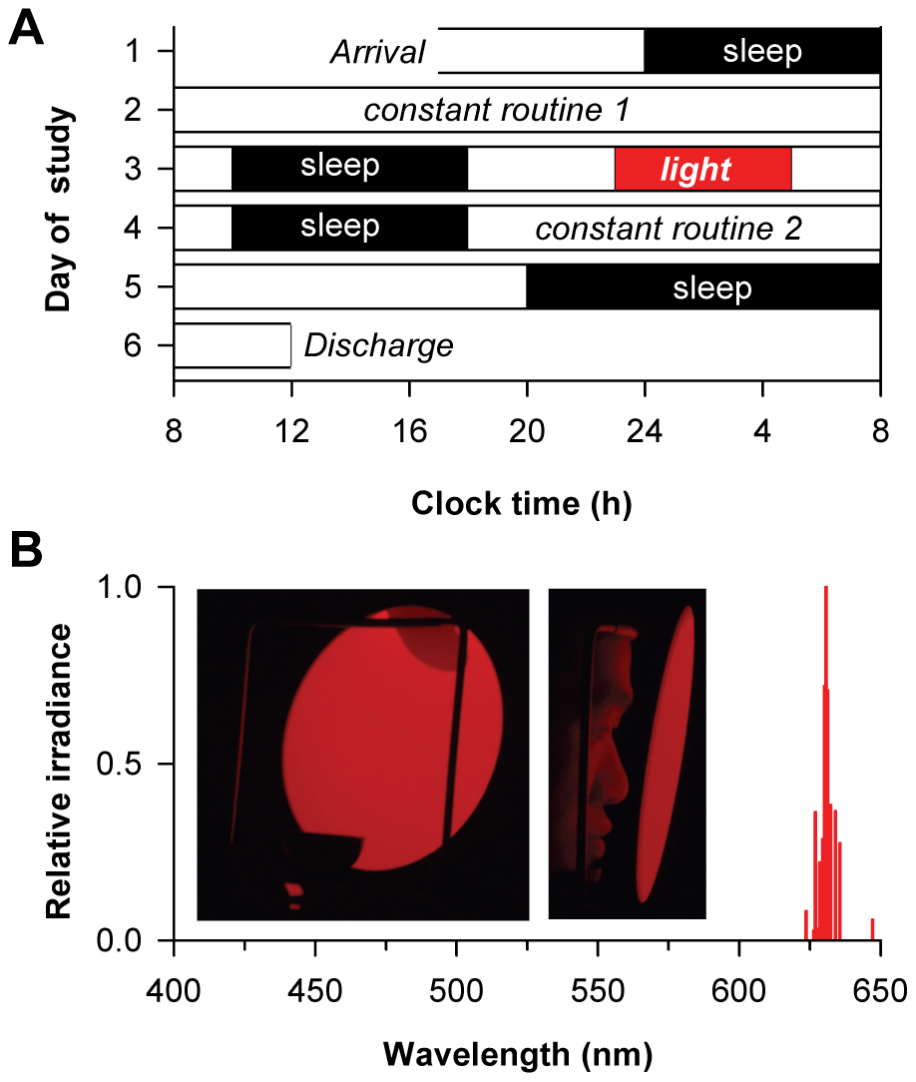
Protocol for assessing circadian phase shift responses and melatonin suppression. (**A**) Subjects took part in a 6-day laboratory study. Circadian rhythms were assessed using constant routine (CR) procedures before and after an experimental light exposure session. During the CR procedure, subjects were exposed to <5 lux of ambient light. During the light exposure session, subjects were exposed to 6 h of continuous red light (631 nm, 13 log photons cm^−2^ s^−1^), intermittent red light and darkness (∼1 min on, 1 min off), or bright polychromatic white light (2,500 lux; 4000K) starting 1 h before habitual bedtime. (**B**) The narrow-bandwidth red light stimulus was generated using a light-emitting diode and delivered to subjects' eyes using a modified Ganzfeld dome. The spectral emission of the LED stimulus is shown.

**Figure 2:**
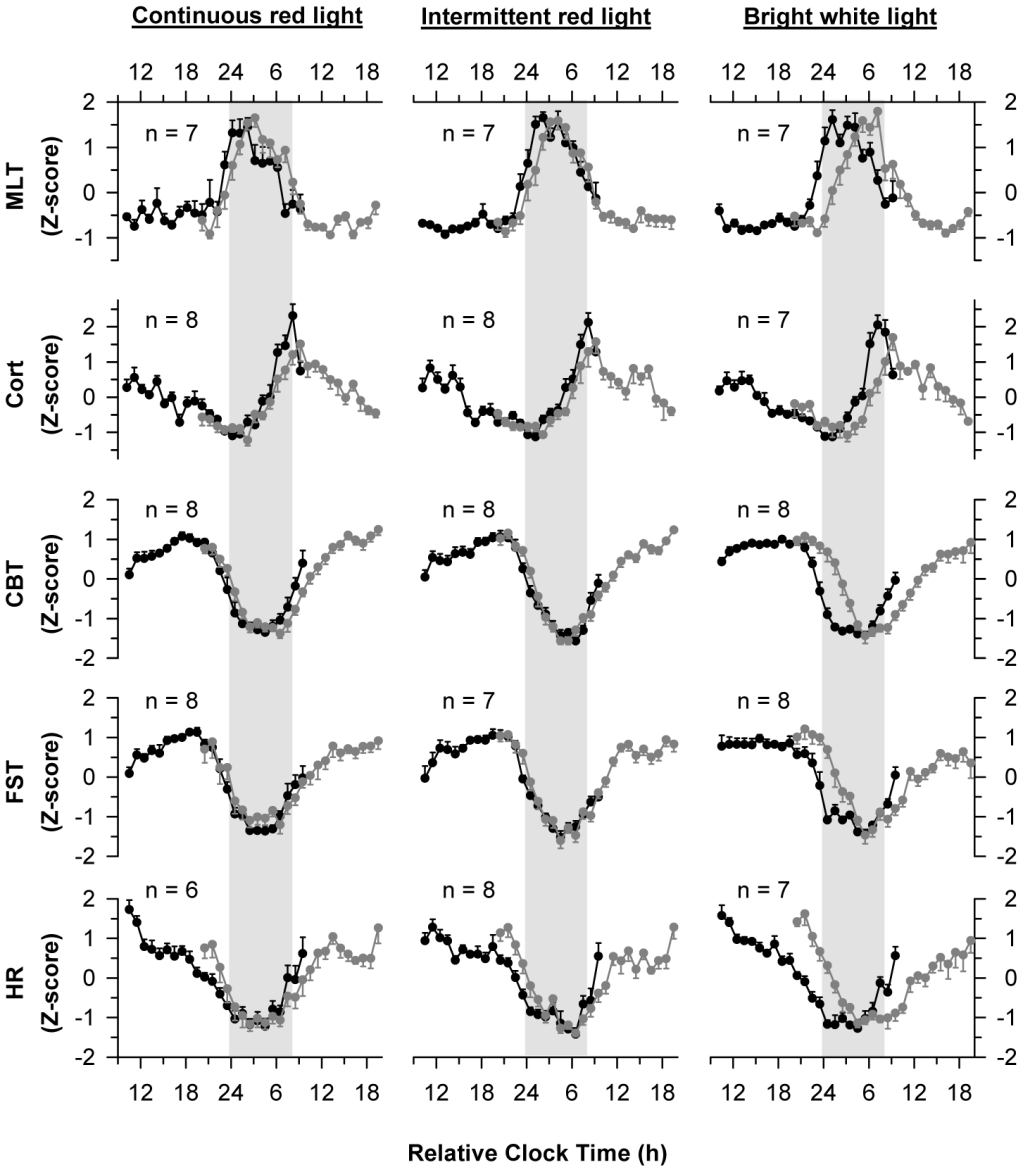
Circadian rhythms assessed before and after exposure to light. Circadian rhythms of melatonin (MLT), cortisol (Cort), core body temperature (CBT), forehead skin temperature (FST), and heart rate (HR) were measured using constant routine procedures. Black traces show rhythms on the day prior to light exposure, and gray traces show rhythms on the day after light exposure. Subjects were exposed to 6 h of continuous red light (left column), intermittent red light and darkness (center column), or bright white light (right column) starting 1 h before habitual bedtime. Results are Z-scored and averaged across subjects with data binned hourly. Vertical gray bars indicate the usual hours of sleep. Since participants had different self-selected bedtimes prior to the study, and study events were timed according to each person's pre-study sleep-wake schedule, analyses are presented using relative clock time, with relative bedtime defined as midnight. Circles with error bars show the mean ± SEM.

**Figure 3:**
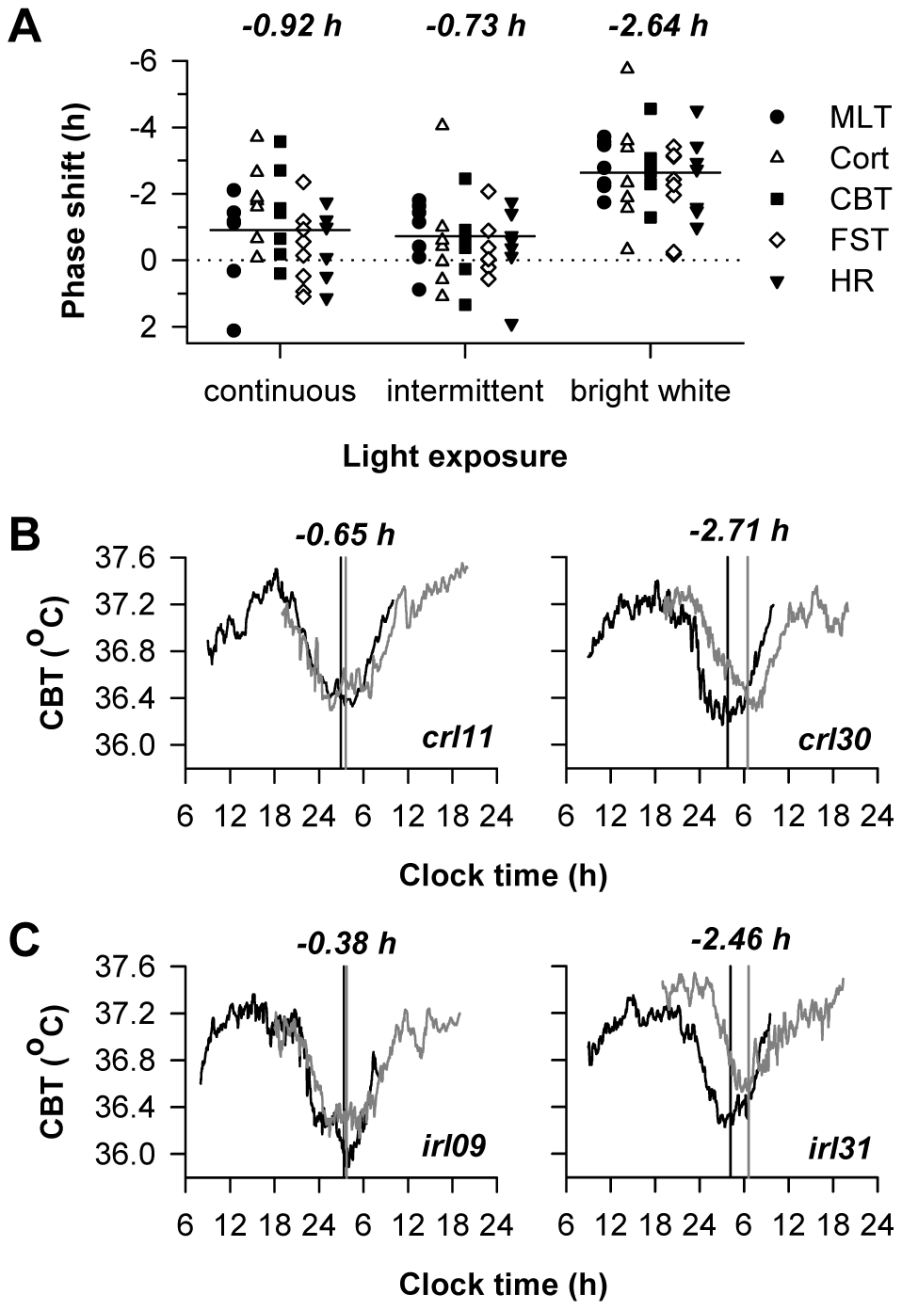
Circadian phase shift responses were similar for exposure to continuous versus intermittent red light. (**A**) Circadian responses are shown for individual subjects exposed to 6 h of continuous red light, intermittent red light and darkness, or bright white light. Circadian phase shifts are shown for melatonin (MLT), cortisol (Cort), core body temperature (CBT), forehead skin temperature (FST), and heart rate (HR). By convention, negative values show phase delay shifts. Horizontal lines show the grand mean for each light exposure condition, with the corresponding values shown at the top of the plot. (**B**) Most subjects exposed to continuous red light exhibited a small resetting response (left), but one participant showed a phase delay shift comparable in magnitude to bright white light exposure (right). (**C**) Likewise, in response to intermittent red light and darkness, circadian phase measured before and after light exposure was similar in most subjects (left); however one subject showed a large phase delay shift (right). In **B** and **C**, representative subjects are shown, with the circadian rhythm of core body temperature (CBT) shown before and after exposure to light (black and gray traces, respectively). Vertical lines show the timing of the fitted minimum for the CBT rhythm. Phase shift values are shown at the top of each plot, and the corresponding subject code is shown on the bottom right.

To assess the contribution of cone photoreceptors to circadian responses, participants were exposed to 6 h of continuous red light or alternating red light and darkness (631 nm, 13 log photons cm^−2^ s^−1^; n = 8 in each group) during the early biological night, as part of a 6-day laboratory study (Fig. 1A–B). An additional group of participants was exposed to bright white light (2,500 lux; n = 8), which served as a positive control for evaluating the relative magnitude of non-visual responses to red light. Circadian rhythms of melatonin, cortisol, core body temperature, forehead skin temperature, and heart rate were assessed on the day before and after the light exposure procedure (Fig. 2). There was a main effect of lighting condition on phase resetting, such that bright white light induced phase shifts that were greater in magnitude compared to either of the red light conditions (*F*_2,20.2_ = 8.76, *P* = 0.002; post-hoc *P*<0.003 for both comparisons). There was no difference in circadian responses to continuous versus intermittent red light (post-hoc *P* = 0.69) assessed across different physiologic measures (Table 1), however, and the average phase shift response to red light was close to an hour in both conditions (Fig. 3A). By comparison, bright white light elicited an average phase shift of −2.64 h, equivalent to resetting the circadian system about 40° of longitude in the westward direction. Although the magnitude of phase shift responses did not differ significantly across physiologic measures (*F*_4,84.0_ = 1.12; *P* = 0.35), there were large inter-individual differences (Wald Z test of between-subject variance; z = 2.41, *P* = 0.016) in phase shift responses (Fig. 3A). Based on the average phase shift response across all available circadian rhythm markers, 6 out of 16 participants exposed to red light showed phase delay shifts greater than an hour (continuous, n = 4; intermittent, n = 2). While most subjects showed little or no circadian response to red light, 2 participants showed phase resetting responses that were similar in magnitude to those who were exposed to bright white light (Fig. 3B–C), demonstrating that red light can elicit robust circadian responses in some individuals.

**Table 1:** Circadian phase shift responses to light (h ± SEM).

Physiologic measure	Continuous red light	Intermittent red light	Bright white light
Salivary melatonin	−0.68±0.54	−0.80±0.37	−2.83±0.29*^†^
Salivary cortisol	−1.55±0.45	−0.54±0.55	−2.69±0.66 ^†^
Core body temperature	−1.24±0.48	−0.48±0.38	−2.82±0.32*^†^
Forehead skin temperature	−0.33±0.41	−0.37±0.33	−2.10±0.45*^†^
Heart rate	−0.41±0.45	−1.23±0.88	−2.53±0.47*

Circadian phase shift responses are shown for 6 h of exposure to continuous red light, intermittent red light and darkness, or bright white light near the onset of melatonin secretion. By convention, negative values indicate phase delay shifts. Using a linear mixed-effects model for comparing phase resetting responses, bright white light elicited a larger response than either red light condition (*P*<0.003). Phase shifts were similar in response to continuous versus intermittent red light (*P* = 0.69), and did not differ across physiologic measures (*P* = 0.35). Data were also analyzed using one-way ANOVA, whereby asterisks (*) indicate significant differences in response to bright white light versus continuous red light, and daggers (^†^) indicate significant differences in response to bright white light versus intermittent red light. Phase resetting did not differ between red light conditions.

**Figure 4:**
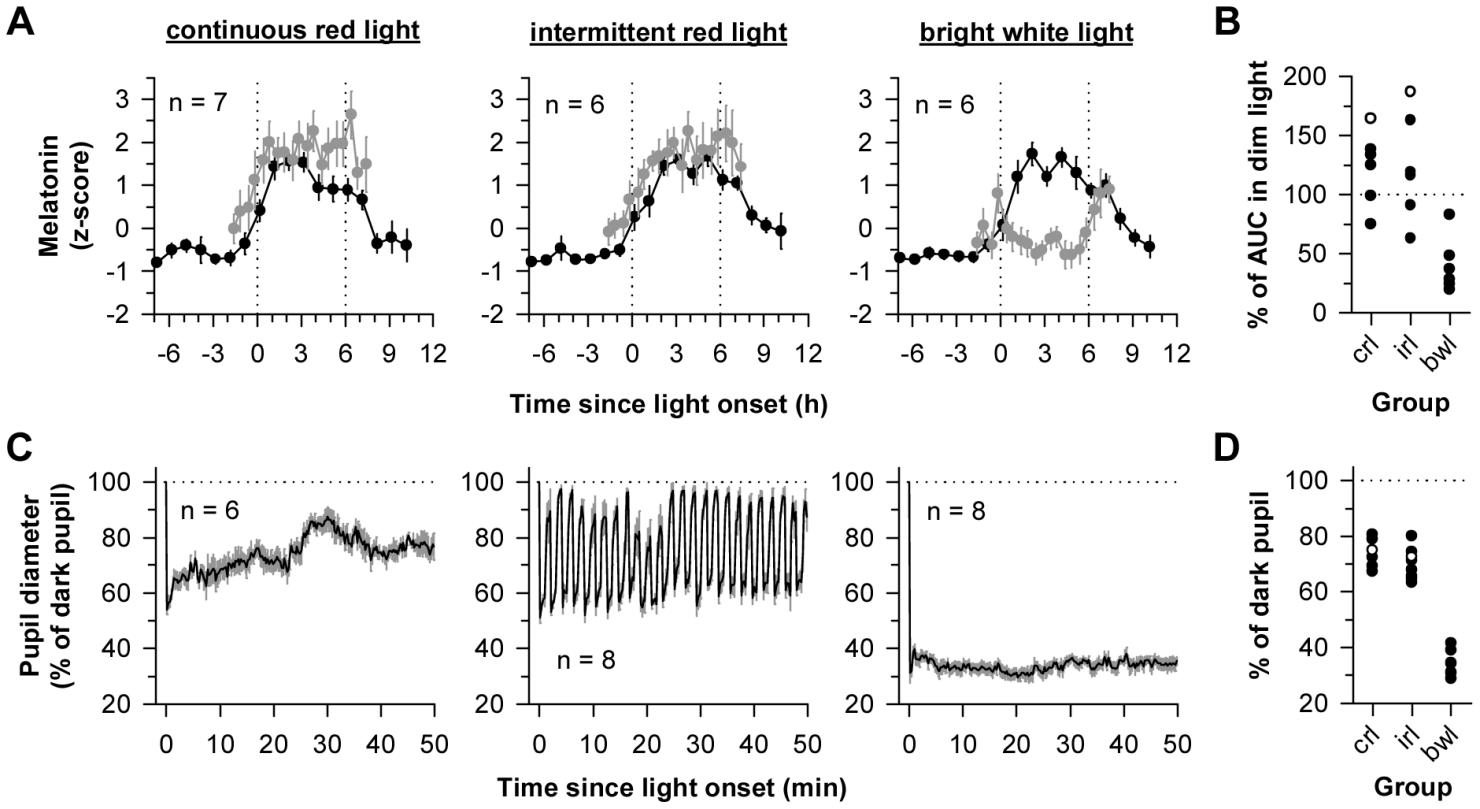
Melatonin levels and pupillary constriction during nocturnal light exposure. (**A**) Melatonin profiles are shown for participants exposed to 6 h of continuous red light (left), intermittent red light and darkness (center), or bright white light (right) near the onset of melatonin secretion. Black traces show the melatonin rhythm on the day prior to light exposure, and gray traces show melatonin on the day of the light exposure session. Melatonin concentrations during light exposure were individually adjusted using Z-score values obtained during the first constant routine procedure. Vertical dotted lines indicate the onset and offset of the light exposure session. (**B**) The area under the curve (AUC) of the melatonin profile during light exposure is shown for each subject, expressed as a percentage of his AUC measured in dim light. Values less than 100% therefore indicate light-induced melatonin suppression, whereas values that exceed 100% indicate that the AUC was higher during the light exposure session relative to the AUC measured in dim light on the previous day. The open circles show responses for subjects *crl30* and *irl31*, who exhibited substantial resetting of circadian rhythms, as shown in Figure 3. (**C**) The pupillary light reflex is shown during the first 50 min of exposure to continuous red light (left), alternating red light and darkness (center), and bright white light (right). (**D**) The median pupillary light response is shown for individual subjects during the 50-min fixed gaze period, expressed relative to the dark pupil. Horizontal dotted lines in **C** and **D** indicate pupil diameter in darkness, and data in **C** are binned at intervals of 15.625 s, corresponding to one-quarter of an intermittent lights-on pulse. In **A** and **C**, the mean ± SEM is shown. In **B** and **D**: crl, continuous red light; irl, intermittent red light; bwl, bright white light.

Next, we compared melatonin levels during the light exposure session with levels measured in dim light on the previous day (Fig. 4A). Exposure to bright white light significantly reduced the area under the curve of the melatonin rhythm relative to continuous or intermittent red light (*F*_2,16_ = 12.60; *P*<0.001; post-hoc *P*<0.001 for both comparisons). Although subjects who were exposed to bright white light showed larger phase shift responses and stronger melatonin suppression than those who were exposed to red light, the degree of melatonin suppression did not associate with the magnitude of circadian phase resetting when assessed across participants in all groups (n = 19, Spearman's rho = −0.23, *P* = 0.34). A reduction in melatonin AUC of greater than 10% was observed in only 2 participants exposed to red light (*crl11*, 24.9%; *irl12*, 36.9%), and the 2 individuals who showed the largest phase shifts (*crl30* and *irl31*; Fig. 3) did not exhibit melatonin suppression (Fig. 4B). By comparison, all participants exhibited pupillary constriction during exposure to red light (Fig. 4C–D). At the beginning of exposure to continuous red light, pupil diameter decreased to about half of the dark-adapted size and then increased gradually over time. During exposure to intermittent red light and darkness, each light pulse elicited a similar response, and pupil diameter dilated toward the dark-adapted state during intervening dark periods. In bright white light, pupillary constriction was strong and sustained, with pupil diameter decreasing to about a third of the size measured in darkness.

## Discussion

Our results show that exposure to alternating red light and darkness in humans did not result in stronger circadian phase resetting or melatonin suppression, as compared to exposure to continuous red light. Hence, contrary to our initial hypothesis, repeatedly activating cone photoreceptors did not enhance circadian or melatonin suppression responses to red light. These findings contrast with results for the pupillary light reflex, which can be enhanced nearly two-fold by exposing the eyes to intermittent light [10]. The present findings are surprising, as it was recently shown that alternating red light and darkness can enhance circadian phase shift responses in transgenic mice carrying human L-cone opsin (*Opn1mw^R^* mice) substituted for the native M-cone opsin [15]. In that study, exposure to 43 min of alternating red light and darkness (1 min on, 2 min off; 644 nm) induced circadian resetting responses that were about an hour greater than exposure to 15 min of continuous light (range, ∼12.5 to 13.5 log photons cm^−2^ s^−1^), even though total illumination time was the same for each condition. In our study, total illumination time in the intermittent light condition was half the amount for subjects exposed to continuous red light. Hence, we cannot rule out the possibility that our results would more closely resemble those obtained in *Opn1mw^R^* mice if we had equated light exposures by total illumination time. It should be noted, however, that phase shift responses were smaller in our subjects compared with *Opn1mw^R^* mice, despite an additional 2 h 45 min of exposure to light, suggesting that results in the mouse model might not translate directly to humans.

To our knowledge, our study is the first to examine the effects of intermittent long-wavelength light on human circadian responses. In prior work, exposure to alternating cycles of bright white light (∼10,000 lux) and dim light (<15 lux) elicited phase shifts similar in magnitude to continuous light [22], even when total illumination time was reduced to 23% of the continuous light exposure [23]. As such, intermittent light was found to be more efficient than continuous light at resetting circadian rhythms when assessed on a per unit time basis. More recently, it was reported that the human circadian system can respond to a series of 2-ms pulses of moderately bright light (473 lux) given over the course of an hour [24]. Therefore, similar to non-visual light responses measured in rodents [25], [26], the circadian system in humans shows a remarkable capacity to temporally integrate light information across intervening periods of darkness. Since we tested only one combination of wavelength, corneal irradiance, and frequency (i.e., 631 nm, 13 log photons cm^−2^ s^−1^, ∼1 min lights on/off), it is possible that other intermittent long-wavelength light stimuli might prove more effective at resetting circadian rhythms and suppressing melatonin.

It is well-established that circadian and melatonin suppression responses are short-wavelength sensitive in the photopic visual range [27]–[30]. In the present study, several subjects showed circadian phase shift responses to red light that were greater than an hour, including 2 individuals who exhibited phase shifts that were comparable in magnitude to responses induced by bright white light. Based on prior work, the red light stimulus we used was outside the range of spectral sensitivity for the intrinsic melanopsin cell response [9], [31], suggesting that visual photoreceptors are capable of resetting the circadian system in humans. These findings are consistent with previous work demonstrating that exposure to 5 hours of red light in the morning over three consecutive days reset the human melatonin rhythm about an hour earlier [32]. More recently it was shown that exposure to low-irradiance 555-nm green light (<12.5 log photons cm^−2^ s^−1^) shifted human circadian rhythms by a greater amount than that expected for a melanopsin-only response [18]. Given that *Opn1mw^R^* mice show significant phase shift responses to long-wavelength red light [15], and mice lacking M-cones show deficits in circadian responses to short-duration light stimuli [17], we hypothesize that our results for red light-induced resetting of circadian rhythms are explained by activation of cone photoreceptors. Rods can elicit non-visual light responses in the photopic visual range in mice [33], however, and transgenic mice with disruption of rod and melanopsin signaling do not reliably entrain to light-dark cycles despite having intact cone function [15], [16]. To assess whether these findings translate to humans, non-visual light responses could be studied in patients with selective loss of either rod or cone photoreceptor function, e.g. individuals with congenital stationary night blindness or congenital achromatopsia.

In the present study, we did not observe a significant melatonin suppression response to long-wavelength red light, which is consistent with prior work demonstrating that only very bright red light exposures (e.g., 630 nm, 18 log photons cm^−2^ s^−1^) suppress melatonin synthesis in humans [27], [34]. Nonetheless, several participants appeared to show circadian phase resetting, and all subjects exhibited pupillary constriction in response to red light. These findings suggest that response thresholds differ across non-visual light responses. Similar to our results, a previous study reported no correlation between the magnitude of phase resetting and the amount of melatonin suppression in response to moderately-bright broad-bandwidth red light [32]. Also, exposure to flashes of broad spectrum white light can reset human circadian rhythms without a reduction in salivary melatonin concentration [24]. Response thresholds for circadian phase shifting and suppression of pineal melatonin also differ substantially in golden hamsters [35], and similar findings have been reported for circadian photic entrainment and pupillary constriction in mice exposed to dim red light [36]. These differences in sensitivity might arise at the level of ipRGCs [37], [38], or could be due to differences in central processing of light information.

An important limitation of the present study is that we did not include a negative control group, and therefore we cannot be sure that the average phase resetting response to red light was greater than the amount of circadian drift in the absence of light exposure. Based on a previous study that used the forced desynchrony protocol to assess circadian period [39], we would expect circadian phase to drift later by ∼20 min over 2 days in most adult males. Since several participants exposed to red light exhibited phase delay responses that exceeded an hour, and 2 individuals displayed phase shift responses that were greater than −2 h (subjects *crl30* and *irl31*; Fig. 3), we consider it unlikely that our results can be attributed solely to circadian drift. To assess accurately the contribution of circadian drift to phase shift responses, it would be necessary to measure circadian period in each subject. Assessing circadian period was beyond the scope of the present study, but the time difference between circadian phase and the sleep cycle (i.e., the phase angle of entrainment) has been shown to correlate with circadian period [40], [41]. It is worth noting, then, that the time difference between the CBT rhythm minimum and wake time was in the normal range in subjects *crl30* and *irl31* (4.2 h and 3.8 h before wake time, respectively), as compared to phase angle measured across all subjects (mean ± SD = 3.8 h±1.1 h). Hence, it is unlikely that the larger-than-average phase shift responses in subjects *crl30* and *irl31* can be explained by an unusually long circadian period relative to other subjects, although we did not test this directly.

Another limitation of our work is that we studied a relatively small number of subjects. We had the statistical power to detect a difference of about 1 hour in circadian phase resetting between groups. We therefore cannot rule out the possibility that, with a much larger group of subjects, a small but significant difference would be found between groups exposed to continuous versus intermittent red light. Our findings suggest, however, that the effect size of such a difference would be very small. We found that individual differences in circadian light responses were substantial, but we did not assess whether such differences are stable and reproducible, as participants completed the protocol only once. Aside from potential trait-like differences in circadian sensitivity, other potential sources of variability for resetting responses include small differences in circadian timing of light exposure, and intrinsic variability in the SCN pacemaker and/or rhythms that were assessed. Also, we did not use a mydriatic agent to dilate subjects' pupils, as one of the goals was to assess whether an intermittent light stimulus could potentially be used to enhance circadian responses in a real-world setting where the pupils are free to respond to light. Therefore, we cannot exclude the possibility that our results for circadian resetting and melatonin suppression were affected by differences in retinal irradiance across light exposure groups, as a result of between-group differences in the magnitude of pupillary constriction [42].

In conclusion, this study provides evidence that cone photoreceptors contribute to circadian phase resetting in some individuals. The basis for inter-individual differences in circadian phase resetting remains to be determined, but our results suggest that some people are very sensitive to red light and can respond to stimuli that target classical visual photoreceptors. In such individuals, exposure to even dim light in the late evening hours could potentially delay the circadian clock and the onset of sleep. Future studies should therefore examine whether individual differences in sensitivity of the circadian system to light modulate relative risk for delayed sleep phase disorder and social jet lag.
